# Author Correction: A double-stranded RNA binding protein enhances drought resistance via protein phase separation in rice

**DOI:** 10.1038/s41467-024-49187-z

**Published:** 2024-06-05

**Authors:** Huaijun Wang, Tiantian Ye, Zilong Guo, Yilong Yao, Haifu Tu, Pengfei Wang, Yu Zhang, Yao Wang, Xiaokai Li, Bingchen Li, Haiyan Xiong, Xuelei Lai, Lizhong Xiong

**Affiliations:** 1 National Key Laboratory of Crop Genetic Improvement, Hubei Hongshan Laboratory, Huazhong Agricultural University, Wuhan, China; 2 Haixia Institute of Science and Technology, Fujian Agriculture and Forestry University, Fuzhou, 350002 China

**Keywords:** Drought, Plant breeding, Agricultural genetics, Plant hormones

Correction to: *Nature Communications* 10.1038/s41467-024-46754-2, published online 21 March 2024

The original version of this Article contained an error in Fig. 7b, in which the colour bar indicating the level of annual precipitation was mislabelled. The correct values for the annual precipitation (mm) should be “1000”, “2000”, “3000”, and “4000”. In addition, the original version of this Article contained an error in the last paragraph of the Results section, which incorrectly read ‘In Southeast China Hills with annual precipitation more than 15000 mm…’. The correct version replaces this sentence with ‘In Southeast China Hills with annual precipitation more than 1500 mm…’. These have been corrected in both the PDF and HTML versions of the Article.

