# Supplementary material for: A double-stranded RNA binding protein enhances drought resistance via protein phase separation in rice
Source: Nat Commun. 2024 Mar 21;15:2514. doi: 10.1038/s41467-024-46754-2 (PMC10957929; doi:10.1038/s41467-024-46754-2)
Supplement: Supplementary file 1 — Supplementary Information [file 41467_2024_46754_MOESM1_ESM.pdf]

A double-stranded RNA binding protein enhances drought resistance via protein phase separation in rice

Wang *et al.*

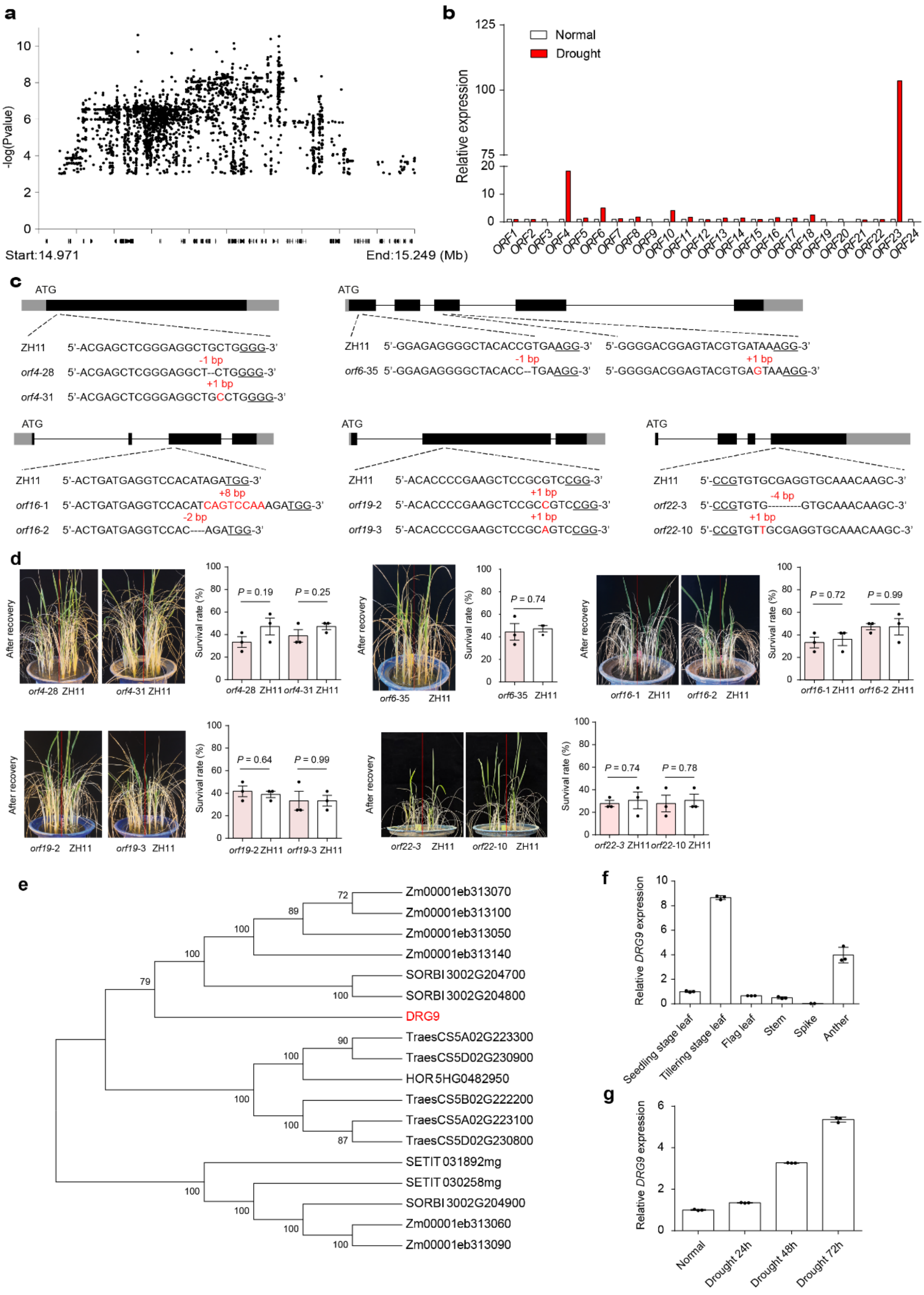

Supplementary Fig. 1. Identification of drought resistance candidate genes. a The candidate gene models within the GPAR_R association region on chromosome 9. **b** Relative expression levels of candidate genes under normal and drought stress. The strongly drought-inducible gene *ORF23* was named *DRG9*. **c** Gene structures and mutation sites in *ORF4*, *ORF6*,

ORF16, *ORF19* and *ORF22*. The sgRNA target sequences are in black and the PAM sites are underlined. **d** Phenotype and survival rate of *orf4*, *orf6*, *orf16*, *orf19* and *orf22* mutants after recovery. Data are mean ± SEM (n = 3 biological replicates). The *P*-value was determined by two-tailed *t*-tests. **e** Phylogenetic tree of DRG9 homologs in monocot crops. Neighbour-joining tree was constructed using MEGA7. **f** The expression patterns of *DRG9* in seedling stage leaf, tillering stage leaf, tillering stage stem, heading stage flag leaf, heading stage spike and heading stage anther. Data are means ± SD (n = 3 biological replicates). **g** The expression level of *DRG9* in seedings under drought stress condition. Data are means ± SD (n = 3 biological replicates). Source data are provided as a Source Data file.

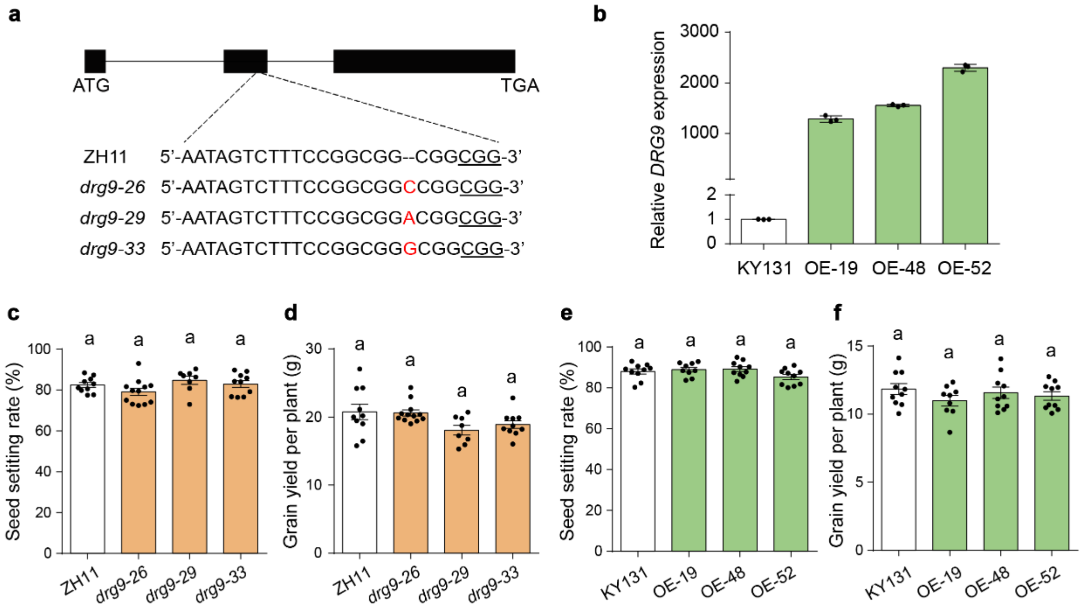

Supplementary Fig. 2. Identification of positive transgenic lines. a Schematic presentation of the *DRG9*. Exons and intron are depicted as black boxes and line, respectively. The sgRNA target sequences are in black and the PAM sites are underlined. **b** The expression of *DRG9* in overexpression lines. Data are means ± SD (n = 3 biological replicates). **c-d** Comparison of seed setting rate, grain yield per plant between ZH11 and *drg9* mutant plant under normal growth conditions in field. Data are mean ± SEM (n = 10, 12, 8, 10 plants). **e-f** Comparison of seed setting rate, grain yield per plant between KY131 and *DRG9* OE plant under normal growth conditions in field. Data are mean ± SEM (n = 10, 9, 10, 10 plants). In **c-f**, the significance of the difference is calculated with a one-way ANOVA analysis-Tukey comparison. Source data are provided as a Source Data file.

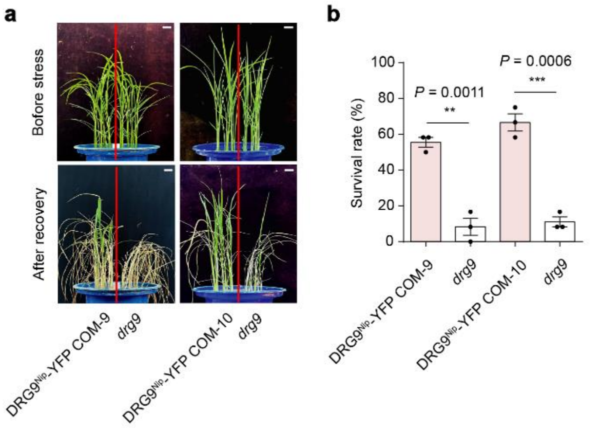

Supplementary Fig. 3. DRG9^{Nip}-YFP transgene can rescue the drought sensitive phenotypes of *drg9*. a Drought resistance of *drg9* mutant compared to DRG9^{Nip}-YFP complemented lines. Scale bar, 2 cm. **b** Survival rates of *drg9* mutant and DRG9^{Nip}-YFP complemented lines after re-watering. Data are means ± SEM (n = 3 biological replicates). Asterisks indicate statistical significance by two-tailed *t*-tests (**P < 0.01, ***P < 0.001). Source data are provided as a Source Data file.

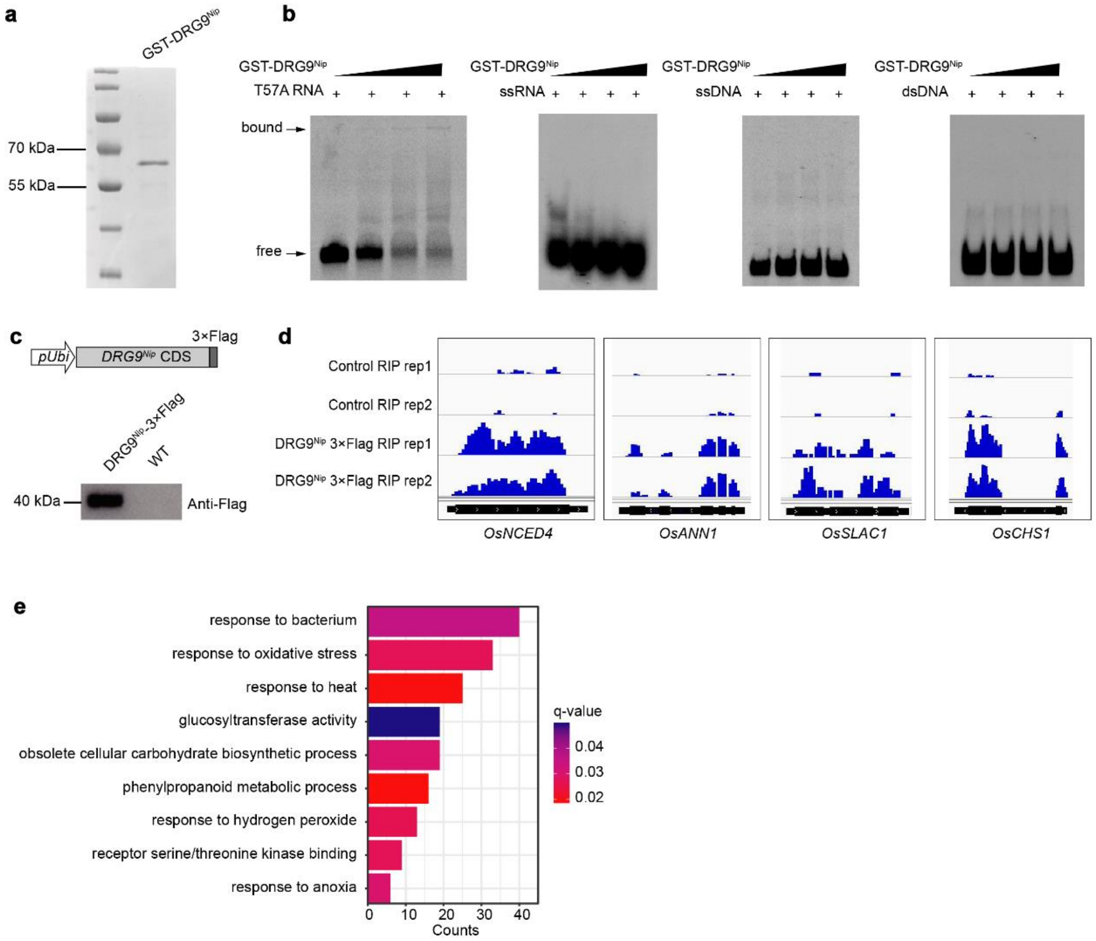

Supplementary Fig. 4. DRG9 binds to RNA *in vitro* and *in vivo*. **a** Coomassie blue staining of GST-tagged DRG9^{Nip} protein. **b** EMSA results showing GST-DRG9^{Nip} binding to T57A RNA, ssRNA, ssDNA and dsDNA probes. **c** Western blot analysis with anti-Flag to detect the DRG9^{Nip}-3×Flag fusion protein in the transgenic plants. **d** IGV genome browser snapshots of normalized coverage of transcripts in RIP-seq. **e** GO analyses of DRG9^{Nip} target transcripts from RIP-seq. In **a-c**, a representative experiment from three independent experiments is shown. Source data are provided as a Source Data file.

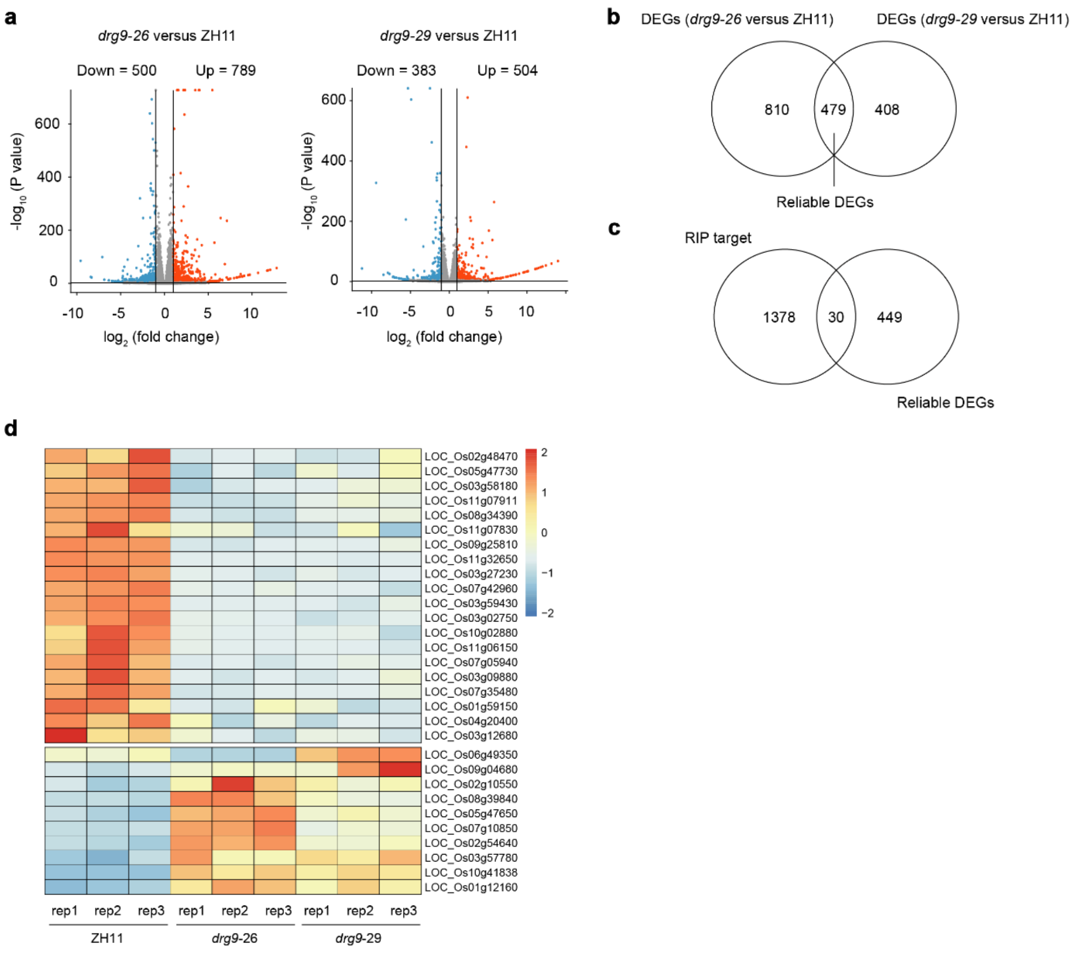

Supplementary Fig. 5. Identification of DEGs between *drg9* and ZH11 under drought conditions. a Volcano plot of DEGs in *drg9* mutants compared to ZH11 under drought stress determined by RNA-seq. Genes with at least two-fold expression change and an adjusted *P* value less than 0.05 were considered as up- or down-regulated genes. **b** Venn diagram showing the overlap of DEGs from *drg9*-26 versus ZH11 and *drg9*-29 versus ZH11. **c** Venn diagram showing the overlap of RIP targets and DEGs. **d** Heat maps showing the relative expression changes (normalized) of 30 identified genes in (c).

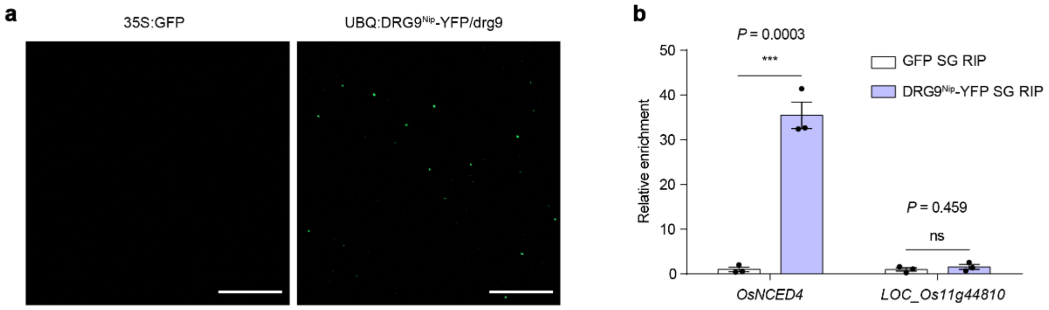

Supplementary Fig. 6. DRG9 bind *OsNCED4* mRNA in SGs. a Purification of SGs from mannitol treated 14-day-old 35S:GFP and UBQ:DRG9^{Nip}-YFP/*drg9* plants. Scale bars = 50 μm. **b** RIP-qPCR validation of the binding of DRG9 to *OsNCED4* mRNAs in SGs. *LOC_Os11g44810*, a DRG9 non-target, was used as the negative control. Two biological replicates were performed with similar results and results from one representative replicate are shown (each in three technical replicates). Asterisks indicate statistical significance by two-tailed *t*-tests (***$P < 0.001$). Source data are provided as a Source Data file.

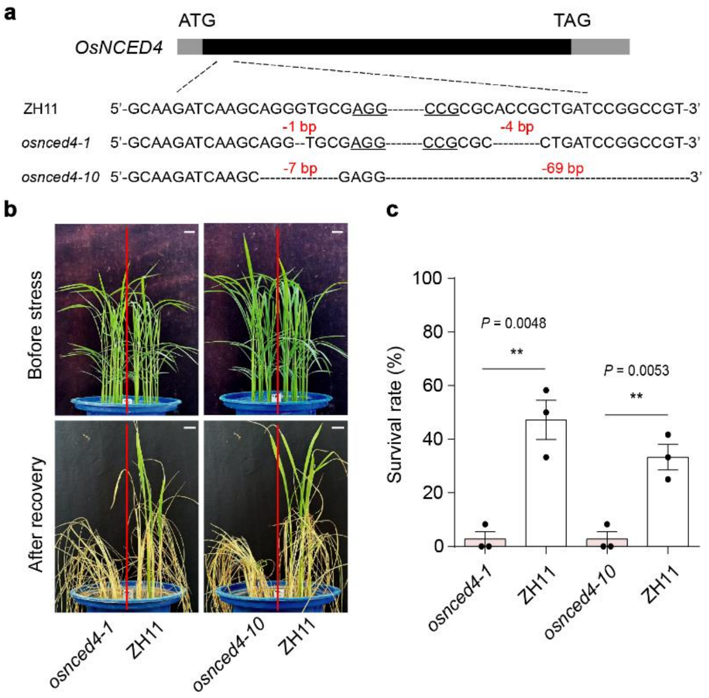

Supplementary Fig. 7. Functional validation of *OsNCED4* in drought resistance. a Schematic presentation of the *OsNCED4*. The sgRNA target sequences are in black and the PAM sites are underlined. **b** Drought resistance of *osnced4* mutant compared to wild-type ZH11. Scale bar, 2 cm. **c** Survival rates of *osnced4* mutant and ZH11 seedlings after re-watering. Data are means ± SEM (n = 3 biological replicates). Asterisks indicate statistical significance by two-tailed *t*-tests (**$P < 0.01$). Source data are provided as a Source Data file.

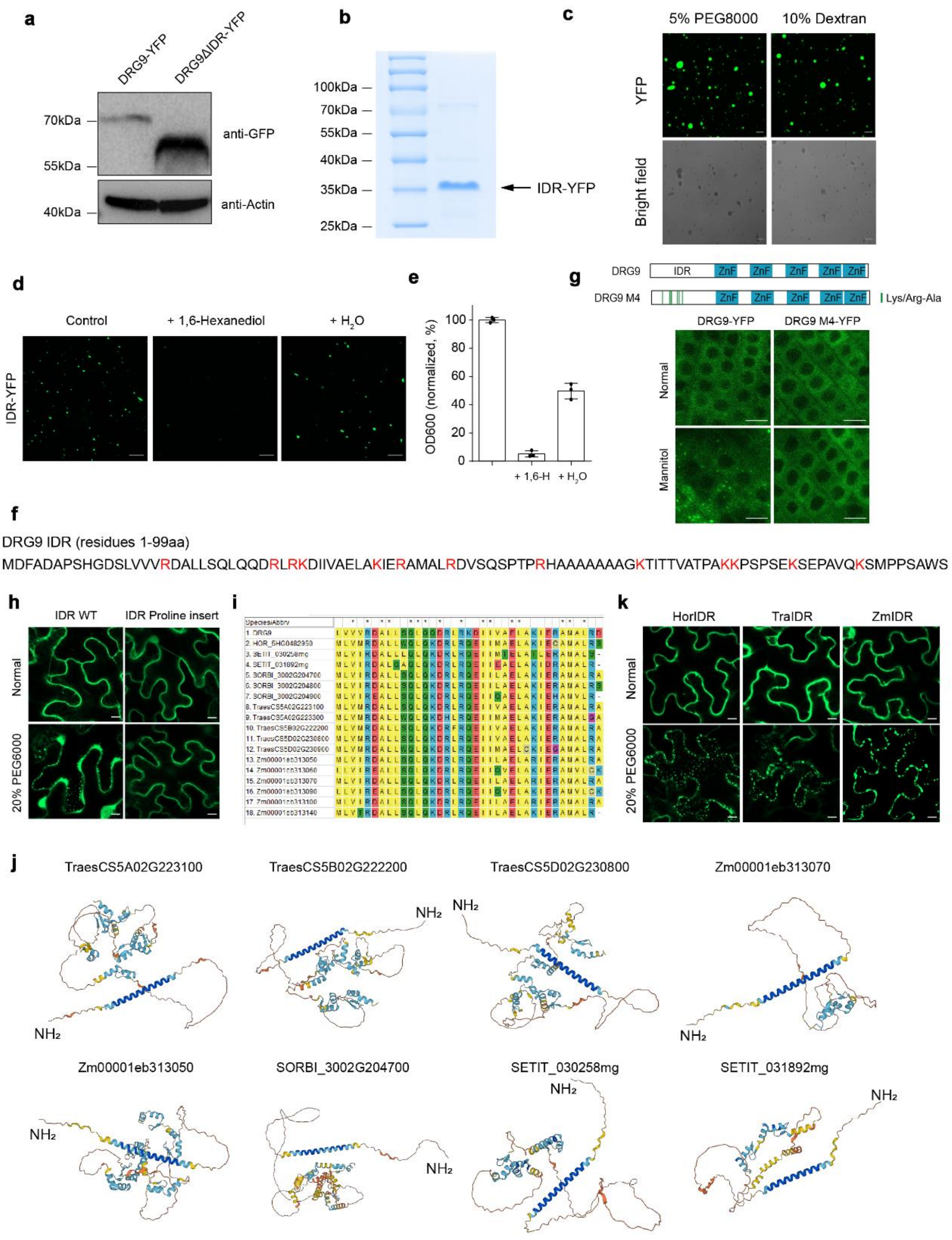

Supplementary Fig. 8. An evolutionarily conserved α-helix is responsible for DRG9 phase separation. **a** Western blots showing the level of DRG9-YFP and DRG9ΔIDR-YFP proteins. **b** Coomassie blue staining of IDR-YFP protein. **c** Representative images showing the LLPS of 12 μM IDR-YFP proteins under indicated conditions (pH 8.0). Scale bars, 10 μm. **d** Addition of 1 volume of 30% 1,6-hexanediol solution disrupts IDR phase separation, Solvent (water) as negative control. Scale bars, 10 μm. **e** Turbidity measurement of solutions shown in (d). Data

are presented as mean ± SEM, (n = 3 replicates). **f** Primary sequence of the DRG9 IDR used in the study. **g** Subcellular localization of rice root tips cells expressing DRG9-YFP or DRG9 M4-YFP before and after mannitol treatment. Scale bars, 10 µm. **h** Confocal microscopic images of tobacco epidermal cells expressing indicated proteins. The cells were treated with or without 20% PEG6000 for 30 min. Scale bars, 10 µm. **i** Multiple sequence alignment of the region corresponding to the α-helix in DRG9 homologs. **j** The structures of DRG9 homologs as predicted by AlphaFold. **k** Confocal microscopic images of tobacco epidermal cells expressing DRG9 homologs (Hor5H0482950, TraesCS5A02G223300, Zm00001eb313050) IDR. The cells were treated with or without 20% PEG6000 for 30 min. Scale bars, 10 µm. In **a-d**, **g**, **h**, **k**, a representative experiment from three independent experiments is shown. Source data are provided as a Source Data file.

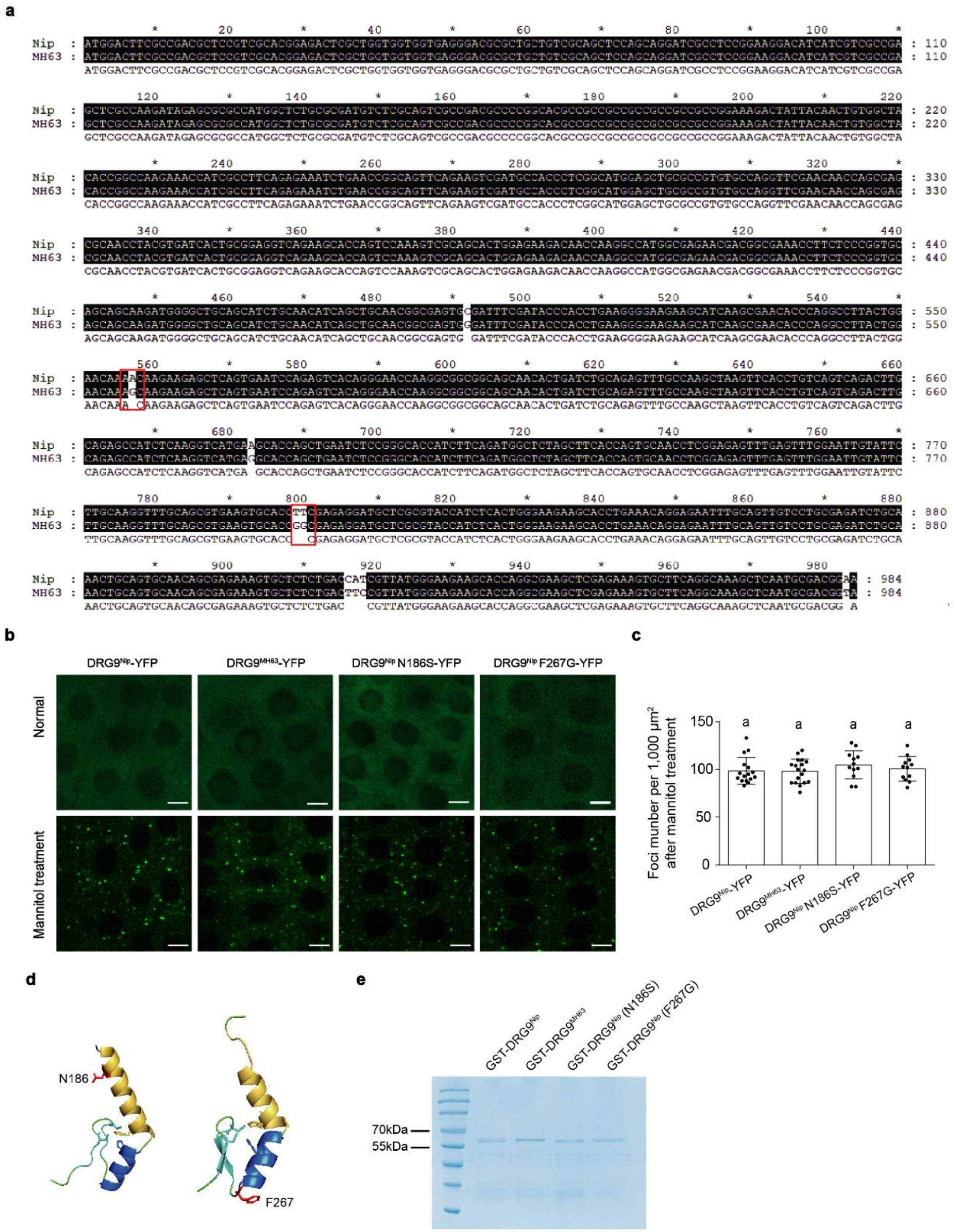

Supplementary Fig. 9. Natural variations in DRG9 leads to changes in amino acids in zinc finger domain. a Comparison of the coding regions of DRG9^{Nip} and DRG9^{MH63}. The red boxes indicate the non-synonymous mutations caused by SNP0823/T>G, SNP0824/T>G and SNP1066/A>G. **b** Subcellular localization of rice root tips cells expressing DRG9^{MH63}-YFP, DRG9^{Nip} N186S-YFP, DRG9^{Nip} F267G-YFP before and after mannitol treatment. Scale bars, 10 μm. **c** Quantification of DRG9^{Nip}-YFP, DRG9^{MH63}-YFP, DRG9^{Nip}-YFP N186S and DRG9^{Nip}

F267G-YFP foci in (b). Data represent the mean ± SD. (n = 16, 18, 12, 12). The significance of the difference is calculated with a one-way ANOVA analysis-Tukey comparison. **d** Structure of zinc finger domain of $DRG9^{Nip}$ as predicted by AlphaFold. **e** Coomassie blue staining of GST-tagged $DRG9^{Nip}$, $DRG9^{MH63}$, $DRG9^{Nip}$ (N186S), $DRG9^{Nip}$ (F267G) protein. In **b**, **e**, a representative experiment from three independent experiments is shown. Source data are provided as a Source Data file.

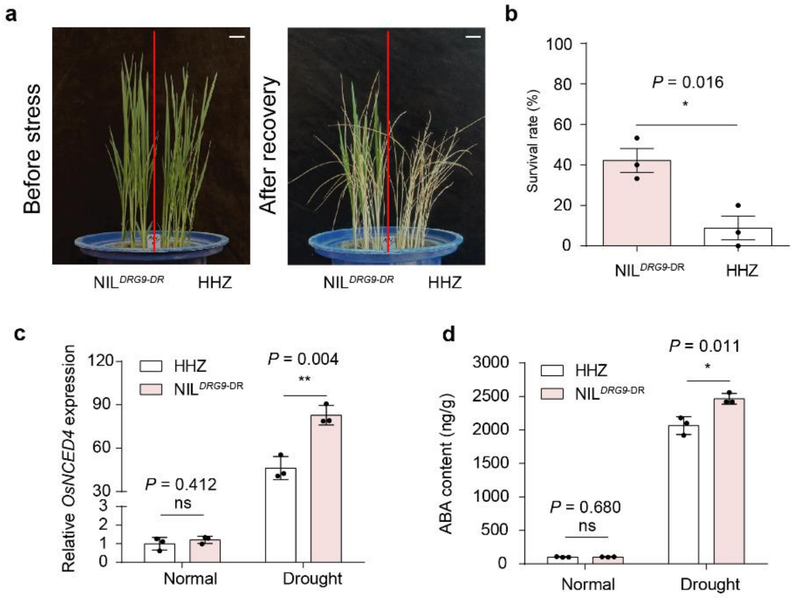

Supplementary Fig. 10. NIL$^{DRG9\text{-DR}}$ exhibited enhanced resistance to drought stress at the seedling stage compared to HHZ. a Drought resistance of NIL$^{DRG9\text{-DR}}$ compared to HHZ. Scale bar, 2 cm. **b** Survival rates of NIL$^{DRG9\text{-DR}}$ compared to HHZ seedlings after re-watering. Data are means ± SEM (n = 3 biological replicates). **c** Relative mRNA levels of *OsNCED4* in NIL$^{DRG9\text{-DR}}$ and HHZ seedlings under normal and drought stress. Data are means ± SEM (n = 3 biological replicates). **d** ABA content in NIL$^{DRG9\text{-DR}}$ and HHZ seedlings under normal and drought stress. Data are means ± SEM (n = 3 biological replicates). Asterisks indicate statistical significance by two-tailed *t*-tests (*$P < 0.05$, **$P < 0.01$). Source data are provided as a Source Data file.

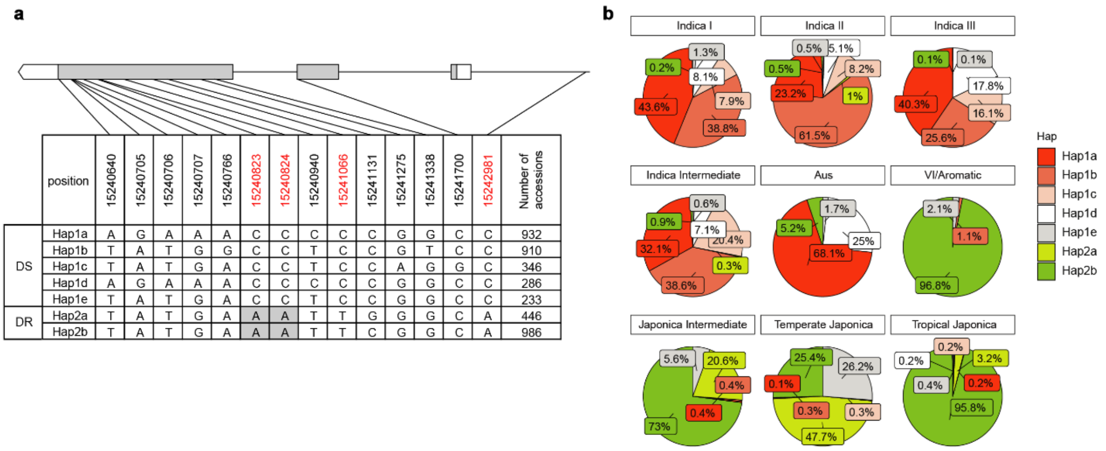

	position	15240640	15240705	15240706	15240707	15240766	15240823	15240824	15240940	15241066	15241131	15241275	15241338	15241700	15242981	Number of accessions
DS	Hap1a	A	G	A	A	A	C	C	C	C	C	G	G	C	C	932
	Hap1b	T	A	T	G	G	C	C	T	C	C	G	T	C	C	910
	Hap1c	T	A	T	G	A	C	C	T	C	C	A	G	G	C	346
	Hap1d	A	G	A	A	A	C	C	C	C	C	G	G	C	C	286
	Hap1e	T	A	T	G	A	C	C	T	C	C	G	G	C	C	233
DR	Hap2a	T	A	T	G	A	A	A	T	T	G	G	G	C	A	446
	Hap2b	T	A	T	G	A	A	A	T	T	C	G	G	C	A	986

Supplementary Fig. 11. Haplotype analysis of *DRG9* in natural population. a Haplotypes of *DRG9* in natural population. The four SNPs identified by GWAS are marked in red. SNP0823 and SNP0824 encoding F267 are labeled in grey boxes. The number of varieties for each haplotype is shown in the right column. **b** Distribution of *DRG9* haplotypes in different subpopulations.

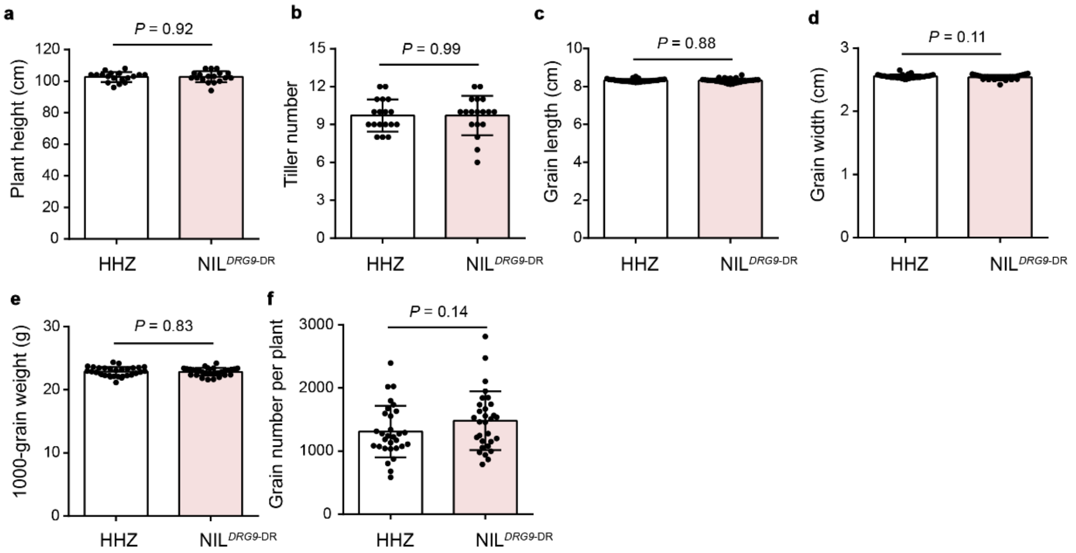

Supplementary Fig. 12. Comparison of agronomic traits between the HHZ and NIL$^{DRG9\text{-}DR}$ plants. a-b Plant height and tiller number of HHZ and NIL$^{DRG9\text{-}DR}$ plants under normal conditions in the field. Data are means ± SD (n = 18/18 plants). **c-f** Grain length, grain width, 1000-grain weight, and grain number per plant of HHZ and NIL$^{DRG9\text{-}DR}$ plants under normal conditions in the field. Data are means ± SD (n = 30/30 plants). The *P*-value was determined by two-tailed *t*-tests. Source data are provided as a Source Data file.
