# Supplementary material for: A double-stranded RNA binding protein enhances drought resistance via protein phase separation in rice
Source: Nat Commun. 2024 Mar 21;15:2514. doi: 10.1038/s41467-024-46754-2 (PMC10957929; doi:10.1038/s41467-024-46754-2)
Supplement: Supplementary file 3 — Description of Additional Supplementary Files [file 41467_2024_46754_MOESM3_ESM.pdf]

Description of Additional Supplementary Files

Supplementary Data 1. FPKM of 24 genes in the candidate region under normal and drought conditions.

Supplementary Data 2. The accessions used for DRG9 expression analyze.

Supplementary Data 3. List of DRG9 binding transcripts from RIP-seq.

Supplementary Data 4. List of DEGs from RNA-seq.

Supplementary Data 5. Distribution of DRG9 haplotypes in different rice sub populations.

Supplementary Data 6. The proportions of DRG9 alleles in lowland rice and upland rice.

Supplementary Data 7. The distribution of DRG9 alleles in China.

Supplementary Data 8. The proportions of DRG9 alleles and annual precipitation in China.

Supplementary Table 9. Primers used in this study.
