# Supplementary material for: A double-stranded RNA binding protein enhances drought resistance via protein phase separation in rice
Source: Nat Commun. 2024 Mar 21;15:2514. doi: 10.1038/s41467-024-46754-2 (PMC10957929; doi:10.1038/s41467-024-46754-2)
Supplement: Supplementary file 13 — Reporting Summary [file 41467_2024_46754_MOESM13_ESM.pdf]

Corresponding author(s): Xuelei Lai
Lizhong Xiong

Last updated by author(s): Mar 2, 2024

Reporting Summary

Nature Portfolio wishes to improve the reproducibility of the work that we publish. This form provides structure for consistency and transparency in reporting. For further information on Nature Portfolio policies, see our Editorial Policies and the Editorial Policy Checklist.

Statistics

For all statistical analyses, confirm that the following items are present in the figure legend, table legend, main text, or Methods section.

n/a	Confirmed	
☐	☒	The exact sample size (*n*) for each experimental group/condition, given as a discrete number and unit of measurement
☐	☒	A statement on whether measurements were taken from distinct samples or whether the same sample was measured repeatedly
☐	☒	The statistical test(s) used AND whether they are one- or two-sided<br>*Only common tests should be described solely by name; describe more complex techniques in the Methods section.*
☒	☐	A description of all covariates tested
☒	☐	A description of any assumptions or corrections, such as tests of normality and adjustment for multiple comparisons
☐	☒	A full description of the statistical parameters including central tendency (e.g. means) or other basic estimates (e.g. regression coefficient) AND variation (e.g. standard deviation) or associated estimates of uncertainty (e.g. confidence intervals)
☐	☒	For null hypothesis testing, the test statistic (e.g. *F*, *t*, *r*) with confidence intervals, effect sizes, degrees of freedom and *P* value noted<br>*Give P values as exact values whenever suitable.*
☒	☐	For Bayesian analysis, information on the choice of priors and Markov chain Monte Carlo settings
☒	☐	For hierarchical and complex designs, identification of the appropriate level for tests and full reporting of outcomes
☐	☒	Estimates of effect sizes (e.g. Cohen's *d*, Pearson's *r*), indicating how they were calculated

Our web collection on statistics for biologists contains articles on many of the points above.

Software and code

Policy information about availability of computer code

Data collection

For RT-qPCR and RIP-qPCR: Applied Biosystems 7500 Fast Real-Time PCR System.
For RNA-seq and RIP-seq: Illumina HiSeq-PE150.
For Microscopy images: Zeiss LSM980 confocal microscope.
For protein concentration: KAIAO K5600 MicroSpectrophotometer.
For plant growth phenotype images: Nikon D7500 Camera.
For EMSA: FUJIFILM FLA-5100 and FUJIFILM FLA-9000
For ABA content measurement: AB SCIEX Triple QuadTM 5500 System

Data analysis

MEGA7 were used for protein phylogenetic tree construction.
Microsoft Excel 2019 and GraphPad Prism 6 were used for statistical analysis of biological data.
Zeiss ZEN software was used to process microscopy images.
AlphaFold and PyMOL (v2.5.2) were used for protein structure analysis.
For RNA-seq, The raw reads were filtered by fastp (v.0.20.1) with default parameters. Then reads were mapped to the rice genome (Nipponbare MSU 7.0) using HISAT2 (v.2.2.1) with default parameters. The read numbers mapped to each gene were counted using featureCounts (v1.5.0) with default parameters. The differential expression analysis was performed using DESeq2 R package (v1.30.1). Genes with |log2(fold change)| > 1 and adjusted P-value < 0.05 were defined as DEGs.
For RIP-seq, The raw reads were filtered by fastp (v.0.20.1) with default parameters. Then reads were mapped to the rice genome (Nipponbare MSU 7.0) using HISAT2 (v.2.2.1) with default parameters. The read numbers mapped to each gene were counted using featureCounts (v1.5.0) with default parameters. The differential expression analysis was performed using DESeq2 R package (v1.30.1). We defined the enriched RNAs in DRG9 RIP versus control as the RNAs showing at least 4-fold enrichment log2(fold change) > 2 and an adjusted P-value less than 0.05.

For manuscripts utilizing custom algorithms or software that are central to the research but not yet described in published literature, software must be made available to editors and reviewers. We strongly encourage code deposition in a community repository (e.g. GitHub). See the Nature Portfolio guidelines for submitting code & software for further information.

Data

Policy information about availability of data

All manuscripts must include a data availability statement. This statement should provide the following information, where applicable:

- Accession codes, unique identifiers, or web links for publicly available datasets
- A description of any restrictions on data availability
- For clinical datasets or third party data, please ensure that the statement adheres to our policy

The RNA-seq data generated in this study have been deposited to the NCBI SRA database under accession code PRJNA1020189. The RIP-seq data generated in this study have been deposited to the NCBI SRA database under accession code PRJNA1063606. The orthologs of maize, wheat, and barley were downloaded from Ensembl plants (http://plants.ensembl.org/Oryza_sativa/Gene/Compara_Ortholog?db=core;g=Os09g0421700;r=9:15240448-15242482;t=Os09t0421700-01). The predicted protein structures were downloaded from AlphaFold database (https://www.alphafold.ebi.ac.uk/). The natural variation information of rice accessions were obtained from 3K Rice Genome Project (https://snp-seek.irri.org/) and RiceVarMap (http://ricevarmap.ncpgr.cn/). The predicted protein structures were downloaded from AlphaFold database (https://www.alphafold.ebi.ac.uk/) with a UniProt accession codes Q69P58 (DRG9), A0A3B6KIZ1 (TraesCS5A02G223100), A0A3B6LMI5 (TraesCS5B02G222200), A0A3B6MSJ5 (TraesCS5D02G230800), A0A804QAH2 (Zm00001eb313070), C0P658 (Zm00001eb313050), C5XCW1 (SORBI_3002G204700), K3ZUH7 (SETIT_030258mg), K3ZZ60 (SETIT_031892mg). Materials used in this study are available upon request. Source data are provided with this paper.

Research involving human participants, their data, or biological material

Policy information about studies with human participants or human data. See also policy information about sex, gender (identity/presentation), and sexual orientation and race, ethnicity and racism.

Reporting on sex and gender	N/A
Reporting on race, ethnicity, or other socially relevant groupings	N/A
Population characteristics	N/A
Recruitment	N/A
Ethics oversight	N/A

Note that full information on the approval of the study protocol must also be provided in the manuscript.

Field-specific reporting

Please select the one below that is the best fit for your research. If you are not sure, read the appropriate sections before making your selection.

☒ Life sciences ☐ Behavioural & social sciences ☐ Ecological, evolutionary & environmental sciences

For a reference copy of the document with all sections, see nature.com/documents/nr-reporting-summary-flat.pdf

Life sciences study design

All studies must disclose on these points even when the disclosure is negative.

Sample size

For drought testing at the seedling stage, more than 3 replicates were selected and used to calculate survival rate. For the drought testing at the reproductive stage in pot, more than 5 samples for each genotype were randomly selected and used for quantification.
Three biological replicates were performed for RNA-seq and two biological replicate was performed for RIP-seq in this study. For RT-qPCR and

	RIP-qPCR, three biological replicates were sampled.<br>Sample size was determined based on our previous experience and from other similar studies. Sample size and statistical method (two sided Student's t-test and Tukey's multiple comparisons test with one-way ANOVA) were described in each figure legend.
Data exclusions	No data were excluded from analysis.
Replication	All experiments were repeated at least 2 times, and similar results were obtained.
Randomization	The plant materials were grown in the same condition and collected randomly, then allocated into experimental groups based on their genotypes. For plant phenotype examination, wild type, mutant and complement lines were randomly selected.
Blinding	Blinding was not relevant to our study. Due to the transgenic materials with different genotypes which should be independently labeled to avoid confusion with each other, making blinding impossible. Therefore, Investigators were not blinded because these kinds of experiments did not generally need blinding.

Reporting for specific materials, systems and methods

We require information from authors about some types of materials, experimental systems and methods used in many studies. Here, indicate whether each material, system or method listed is relevant to your study. If you are not sure if a list item applies to your research, read the appropriate section before selecting a response.

Materials & experimental systems

n/a	Involved in the study	
☐	☒	Antibodies
☒	☐	Eukaryotic cell lines
☒	☐	Palaeontology and archaeology
☒	☐	Animals and other organisms
☒	☐	Clinical data
☒	☐	Dual use research of concern
☐	☒	Plants

Methods

n/a	Involved in the study	
☒	☐	ChIP-seq
☒	☐	Flow cytometry
☒	☐	MRI-based neuroimaging

Antibodies

Antibodies used	anti-Flag: Mouse monoclonal antibody, Sigma (F3165). Dilution 1;5000<br>anti-GFP: Rabbit polyclonal antibody, Abclonal (AE011). Dilution 1;5000<br>anti-Actin: Mouse monoclonal antibody, Abclonal (AC009). Dilution 1;5000<br>anti-Rabbit IgG: HRP Goat Anti-Rabbit IgG (H+L), Abclonal (AS014). Dilution 1;10000<br>anti-Mouse IgG: HRP Goat Anti-Mouse IgG (H+L), Abclonal (AS003). Dilution 1;10000
Validation	anti-Flag, Sigma (F3165): https://www.sigmaaldrich.cn/CN/zh/product/sigma/f3165<br>anti-GFP, Abclonal (AS014): https://abclonal.com.cn/catalog/AE011<br>anti-Actin, Abclonal (AC009): https://abclonal.com.cn/catalog/AC009<br>anti-Rabbit IgG, Abclonal (AS014): https://abclonal.com.cn/catalog/AS014<br>anti-Mouse IgG, Abclonal (AS003): https://abclonal.com.cn/catalog/AS003

Dual use research of concern

Policy information about dual use research of concern

Hazards

Could the accidental, deliberate or reckless misuse of agents or technologies generated in the work, or the application of information presented in the manuscript, pose a threat to:

No	Yes	
☒	☐	Public health
☒	☐	National security
☒	☐	Crops and/or livestock
☒	☐	Ecosystems
☒	☐	Any other significant area

Experiments of concern

Does the work involve any of these experiments of concern:

No	Yes	
☒	☐	Demonstrate how to render a vaccine ineffective
☒	☐	Confer resistance to therapeutically useful antibiotics or antiviral agents
☒	☐	Enhance the virulence of a pathogen or render a nonpathogen virulent
☒	☐	Increase transmissibility of a pathogen
☒	☐	Alter the host range of a pathogen
☒	☐	Enable evasion of diagnostic/detection modalities
☒	☐	Enable the weaponization of a biological agent or toxin
☒	☐	Any other potentially harmful combination of experiments and agents
